# Cognitive Reflection Enhances Rationality Without Changing the Underlying Cognitive Processes

**DOI:** 10.3390/bs16060858

**Published:** 2026-05-27

**Authors:** Andreas Glöckner, Marc Jekel

**Affiliations:** Department of Psychology, University of Cologne, 50931 Köln, Germany; marc.jekel@uni-koeln.de

**Keywords:** dual process, intuition, deliberation, cognitive reflection, rationality, coherence-based reasoning

## Abstract

This study (*N* = 249) examines the influence of cognitive reflection on rational decision making in tasks that require the—potentially rapid—integration of multiple pieces of information but are not designed such that intuitive (System 1) responses mislead people. Cognitive reflection was measured using the Cognitive Reflection Test (CRT). Choice behavior was analyzed in 250 probabilistic inference tasks and 16 risky-decision tasks completed by each participant. In both tasks, individuals with higher CRT scores made more rational choices. This superior performance was not attributable to qualitative differences in cognitive processes. For individuals low and high in cognitive reflection, the same Parallel Constraint Satisfaction Model best explained their choice behavior. High-reflective individuals appeared to use the same coherence-based processes more efficiently and consistently. The absence of qualitative process differences across individuals varying in their tendency to engage in deliberate processing supports an integrative account of dual-process models, particularly those grounded in interactive activation frameworks.

## 1. Introduction

In various domains of cognition, scholars have argued that processes are best described by an interplay of two qualitatively distinct types of processing. This view has led to the development of numerous dual-process models (see De Neys, 2023; Evans, 2008; Hochman, 2024, for reviews). Dual-process models assume that people possess two systems of thinking: System 1, also referred to as intuitive thinking, and System 2, also referred to as reflective thinking (Evans, 2003, 2008; Stanovich, 2011). In the domain of judgment and decision making, for example, both theoretical and empirical work indicate that individuals can rely on their intuitions when making decisions (Hogarth, 2010). Intuition is thereby often understood as a cognition or feeling that one should choose or avoid an option without knowing exactly why or where this inclination originates from. This kind of decision making is typically contrasted with a supposedly qualitatively different process in which individuals are fully aware of how they arrived at their decisions.

It has been shown that individuals differ in their degree of cognitive reflection (e.g., Frederick, 2005), that is, in their tendency to activate deliberate (System 2) processes or rely mainly on intuitive (System 1) processes. Cognitive reflection is measured with biasing tasks, in which superficial or heuristic cues suggest an incorrect response, whereas attending to relevant or rational cues leads to the correct solution. Much of the work on cognitive reflection and choice has also relied on such biasing tasks. In the current research, we investigate how interindividual differences concerning cognitive reflection are associated with choice behavior in non-biasing decision tasks, that is, tasks that do not contain misleading heuristic cues.

At a theoretical level, we aim to clarify the cognitive processes associated with System 1 and System 2. For tasks without biasing cues (e.g., generic probabilistic inferences, or risky choices see below), individuals’ System 1 responses are well predicted by interactive activation neural network models, particularly Parallel Constraint Satisfaction Models (PCS) (Glöckner & Betsch, 2008a; Glöckner et al., 2014, 2024). A recent study manipulating decision mode furthermore showed that the model also accounts for deliberate decision making (Forst & Glöckner, 2026). The instruction to deliberate not only increased choice quality, bringing decisions more in line with rational standards, but also resulted in patterns of response times and confidence ratings that matched PCS model predictions more closely than under the instruction to decide intuitively. This suggests that typical instruction-based inductions of decision mode do not elicit qualitatively different processes (see also Horstmann et al., 2009). This finding speaks against classic dual-process models that postulate inherently qualitative differences between the two assumed kinds of processes. It provides evidence for integrated dual-process models, assuming that System 1 and System 2 processes are not entirely distinct but interact and are based on similar or even the same kind of cognitive processes (details and definition see below).

In the current study, we therefore examine not only choices but also various process measures to assess potential processing differences between persons with higher vs. lower tendencies toward deliberate cognitive reflection.

### 1.1. Classic vs. Integrated Dual-Process Models

The research on dual-process models has examined correlates of System 1 thinking that include, among others, fast, high-capacity, parallel, automatic, associative, and biased thinking, and correlates of System 2 thinking that include slow, capacity-limited, serial, conscious, and normative thinking (Evans & Stanovich, 2013, Table 1, p. 225). However, scholars have argued that System 1 processes do not represent a single, uniform type of process but encompass multiple cognitive processes with distinct properties (Evans, 2008; Glöckner & Witteman, 2010). The same applies to System 2 (Evans, 2008). Consequently, the properties typically attributed to the two systems do not always co-occur. Scholars have also debated how the two systems interact and whether positing two distinct systems is necessary (e.g., Evans, 2008; De Neys, 2023). In this debate, the core assumption of dual-process models—that individuals rely on two qualitatively distinct thinking systems—has been challenged. It has been suggested that the differences between these systems are merely quantitative (e.g., extent of working memory participation and automaticity), and a single-process model or integrated process models could more parsimoniously account for the findings of dual-process research (De Neys, 2021; Hayes et al., 2018; Kruglanski, 2013; Osman, 2004; Stephens et al., 2018, 2020). Perspectives on single-process models and integrated dual-process models vary substantially between researchers. We operationally define integrated dual-process models as models in which the basic information integration process remains the same across (purely) intuitive and (additionally) deliberate decision making, as indicated by the fact that both can be captured by the same cognitive process model and differ only in gradual parameter changes. Note that this does not preclude qualitative differences on other levels such as different subjective experiences (i.e., being aware of the information integration process vs. not). Quite to the contrary, self-reports on these differences can be used to check whether subjectively more intuitive or deliberate decision making is indeed used before investigating whether both can be accounted for by the same process model (Forst & Glöckner, 2026).

Various classes of cognitive processes could serve as the basis for integrated dual-process models. Glöckner and Witteman (2010) suggested the following classification: (a) associative intuition, which summarize memory mechanisms based on simple learning–retrieval processes, including various forms of reinforcement and association learning and retrieval in the form of feelings of liking (e.g., affect heuristic, Finucane et al., 2000) but also activation of previous successful options; (b) matching intuition, which summarizes more complex memory processes based on comparisons of current decision situations with prototypes or exemplars stored in memory (e.g., Dougherty et al., 1999); (c) accumulative intuition, which summarizes the broad class of automatic evidence accumulation and diffusion models that describe rapid information integration in perception (e.g., Busemeyer & Townsend, 1993); and (d) constructive intuition, which summarizes interactive activation models of perception and cognition (e.g., Holyoak & Simon, 1999) that describe (e.g.,) perception as partially automatic construction of consistent mental representations, including accentuation of information to form coherent interpretations, stories, or good shapes (“Gestalt”). The PCS models introduced above belong to this class of processes.

According to a recent integrative conceptual approach (De Neys, 2023), various response tendencies are automatically activated by cues when a person encounters a decision situation. All of these are considered System 1 responses. Cues can trigger irrational or incorrect responses as well as rational or correct responses. The former are referred to as heuristic intuitions—from the perspective of Kahneman and Tversky’s heuristics-and-biases program (Kahneman & Tversky, 1972, 1973), which describes heuristics as mental shortcuts that can lead to biased judgments. The latter are called logical intuitions (De Neys, 2012). These intuitions are assumed to be constantly integrated and, when conflicting, weighted against each other. The underlying monitoring process generates a measure of uncertainty, and deliberation begins only when this uncertainty exceeds a certain threshold.

One possible cognitive implementation of the integration process during monitoring in System 1 is evidence accumulation as an instantiation of accumulative intuition introduced above (Glöckner & Witteman, 2010). In this process, evidence from all cues is randomly sampled (proportionally to their importance) and added competitively in a joint accumulator (e.g., Busemeyer & Townsend, 1993; for a recent dual-process implementation, see Alós-Ferrer, 2018). One could, for example, assume an evidence threshold that must be reached to make a choice and a deliberation threshold that leads to the activation of deliberation when uncertainty reaches a certain level. This could be implemented either with a separate accumulator for uncertainty/ambivalence or by having a criterion that measures whether the accumulated evidence remains over a longer period of time close to zero in the same accumulator.

In the former implementation of the approach suggested by De Neys (2023), deliberation should be activated when the accumulated evidence lies below the deliberation threshold (which then functions as a “lack-of-evidence threshold”) and the decision threshold, thereby preventing a decision based solely on System 1 processing. Decision thresholds and deliberation thresholds vary with task characteristics, such as the importance of the decision, as well as with person characteristics. Individuals who rely more on deliberate and reflective processes adopt wider evidence bounds for their decisions but narrower bounds for activating deliberation. Such individuals, those scoring high on the Cognitive Reflection Test (Frederick, 2005, further details below), should also show higher confidence because they tolerate uncertainty less and reduce it by applying higher evidence thresholds for a decision. These higher thresholds lead to longer response times for individuals high in cognitive reflection. We therefore assume that the decision threshold and evidence threshold are negatively related, since individuals with higher CRT scores require less evidence for activating deliberation (a lower deliberation threshold) and more evidence to make a decision (a higher decision threshold).

Glöckner and Betsch (2008a) proposed a dual-process model that likewise assumes the integration of logical and heuristic cues, but is based on interactive activation processes belonging to the class of constructive intuition. The Parallel Constraint Satisfaction Theory of Decision Making (PCS-DM, Glöckner et al., 2014) proposes that information is stored and processed through interactive activation in neural networks. The model holds that, through spreading activation, individuals activate all cues that are presented in or associated with the decision situation. The activated cues form a temporary network that represents a mental representation of the decision situation. The cues are integrated through coherence structuring and sense making via interactive activation. Interactive activation is a universal principle of cognition applied in perception and many other domains of cognition (McClelland, 2010; McClelland et al., 2014). According to Glöckner and Betsch, deliberation processes are activated only if the coherence in the network remains below a threshold that the individual deems sufficient for the situation at hand. Deliberation serves to double-check but also to support the coherence construction process. In line with default–interventionist dual-process models of decision making (Evans, 2008; Evans & Stanovich, 2013), PCS-DM assumes that people do not fully shift between System 1 and System 2. Instead, System 1 is activated by default. System 2 processes are activated only under certain conditions and used to intervene in, control, support, and—if necessary—correct and debias System 1.

**Figure 1:**
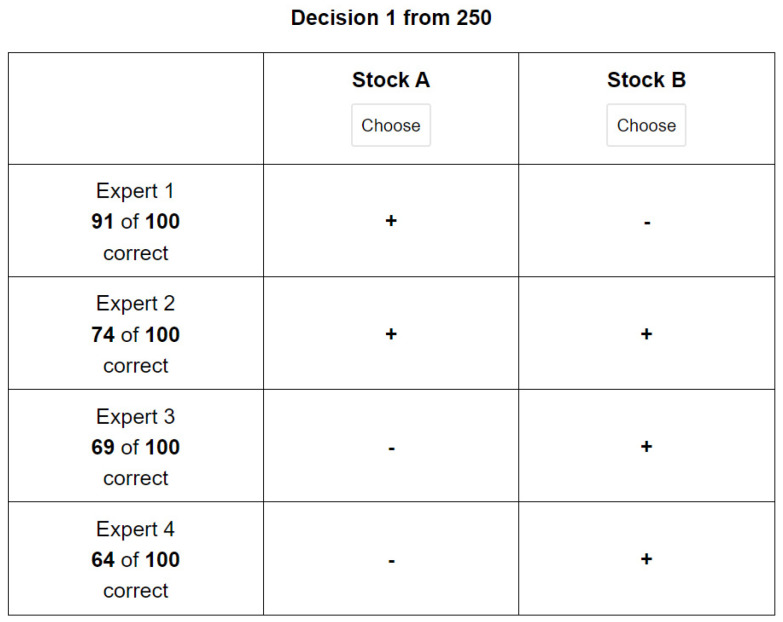
Example probabilistic inference task (translated from German). Plus (minus) means that the cue (i.e., expert) predicts a positive (negative) performance of the stock. The numbers provide the a priori validity of each cue, expressed as relative frequencies.

PCS-DM has been particularly successful in predicting intuitive choice behavior in probabilistic inference tasks (Glöckner et al., 2024). In probabilistic inference tasks, individuals choose among several options based on probabilistic cues (Figure 1). For this class of tasks, it has often been argued that people use fast-and-frugal heuristics to arrive at choices quickly and efficiently (Gigerenzer & Goldstein, 1996). These include, for example, the take-the-best heuristic, according to which individuals retrieve only the most valid (i.e., best) cue, decide based on the prediction of this cue, and ignore the remaining cues. Another example is the equal weight heuristic, which assumes that people ignore cue validities and choose the option supported by the majority of cues. Later research, however, showed that in many situations only a small proportion of people use such strategies. Most participants instead show choice behavior that aligns more closely with the predictions of PCS-DM in intuitive decisions. Somewhat surprisingly, individuals align even more closely with the predictions of PCS-DM when they receive instructions to use a deliberate strategy (Forst & Glöckner, 2026). Hence, initial empirical evidence supports PCS-DM as an integrated dual-process model that can account for both System 1 and System 2 processes.

PCS-DM also has some theoretical advantages over alternative cognitive implementations. PCS-DM avoids a complication of evidence accumulation models, namely the need for two different thresholds (or accumulators). Higher cognitive reflection may manifest in several ways in PCS-DM. Cognitive reflection might lead to more rational choices because (a) individuals high in cognitive reflection are more likely to construct their mental representation based on relevant (as compared to irrelevant) cues and to apply cue weighting that is more in line with rational standards. Relatedly, they (b) might apply cue weighting and integration more efficiently and consistently because they avoid errors and inconsistencies and more efficiently detect contradictions when translating the provided information in each task into their mental representation. Furthermore, they (c) might tend to activate deliberation earlier to double-check the mental representation. This should particularly be the case for tasks in which it is difficult to construct a coherent interpretation. Therefore, decision time might further increase, and awareness of uncertainty (i.e., low confidence) might be further enhanced specifically in these tasks, for which System 1 processes also predict similar patterns (i.e., particularly high response time and low confidence for tasks in which the evidence for both options is about equally strong). The observed response times and confidence patterns across different decision tasks might therefore be more in line with the predictions of PCS-DM for individuals who tend to activate deliberation earlier. That is, one would not necessarily expect main effects on time and confidence but higher correlations between predicted and observed behavior for these participants, consistent with Forst and Glöckner (2026).

### 1.2. Decision Making in Probabilistic Inference Tasks

In this article, we investigate how people use probabilistic information in a standard probabilistic inference task, focusing on the attribute of (ir)rationality to assess whether System 2 processes facilitate the rational integration of probabilistic information according to probability theory. Importantly, in contrast to classic judgment tasks that are typically used to demonstrate biases, these tasks are not constructed to mislead individuals or elicit biases. They consist simply of pieces of information presented in a neutral form that can be used in a rational manner. Hence, these tasks do not require using cognitive reflection to correct for intentionally misleading impulses.

One property of these tasks is that people must integrate information from multiple probabilistic cues to solve them properly. It is unlikely that people use a complex rational-analytic System 2 process for these tasks (i.e., such as naïve Bayes), given the short time individuals typically need to make such decisions (Glöckner & Betsch, 2008b, 2012). Moreover, evidence shows that most participants do not rely on simplifying fast-and-frugal heuristics (i.e., rules of thumb; Gigerenzer et al., 1999) that ignore large parts of the information and that they might apply automatically or deliberately as part of System 1 or 2 (e.g., Ayal & Hochman, 2009; Bröder, 2000; Glöckner et al., 2014; Glöckner & Betsch, 2008b; Heck & Erdfelder, 2017). Researchers have argued that in such tasks most individuals rely on System 1 processes that allow them to integrate information rapidly and efficiently (e.g., Glöckner & Betsch, 2008b, 2012; Glöckner et al., 2014) and that approximate rational solutions very well by weighting cues according to their validity (Jekel et al., 2012).

### 1.3. Previous Findings on Cognitive Reflection

In line with a default–interventionist view on dual-process models, research shows that decisions are strongly influenced by individuals’ tendency to be inclined to engage in deliberate, reflective processes. The Cognitive Reflection Test (CRT) specifically measures individuals’ tendency to override incorrect intuitive responses and engage in further reflection to find a correct answer (Frederick, 2005). Frederick showed that CRT scores are positively related to time preferences (i.e., individuals with higher CRT scores are more patient) as well as to risk preferences (see also Oechssler et al., 2009). Specifically, higher CRT scores are associated with lower risk aversion in the gains domain and with more frequent choices of the expected value-maximizing option (also, e.g., accepting sure losses in the loss domain). Several studies have also shown that CRT scores are inversely related to irrational responses in various classic bias tasks, even after controlling for cognitive ability (e.g., intelligence) and other factors (e.g., Liberali et al., 2012; Toplak et al., 2014). Oechssler et al. (2009), for example, found that individuals with low (compared to high) CRT scores were more prone to the conjunction fallacy and conservatism but did not show stronger anchoring effects.

There are, however, also noteworthy studies that have investigated the effects of CRT in tasks that involve the integration of probabilities related to the paradigms we use in the current study. Specifically, Lesage et al. (2013) showed that persons with high CRT scores performed better in various versions of a classic base-rate neglect task, in which a highly diagnostic mammography result conflicts with a low base rate for the disease. In this task, participants typically do not sufficiently take base-rate information into account because they overlook it or consider it less relevant. The correlation between CRT and performance was particularly strong for presentation formats in which information was easier to process, specifically when it was presented in absolute frequencies. Participants with higher CRT scores could perform the task even under working memory load conditions, indicating automatic or intuitive processing. In a similar vein, Sirota and Juanchich (2011) showed that persons with high CRT scores achieved higher performance in a base-rate task presented in a frequency format. Given these results, one might expect that CRT also leads to higher performance in probabilistic inference tasks. The important difference between the tasks is, however, that in the probabilistic inference task it is clear that all cues are relevant and no contextual factors direct attention away from them. In base-rate neglect tasks, in contrast, the narrative context can lead participants to focus on the descriptive information and overlook the base rates.

Whether individuals’ tendency to reflect on information and engage System 2 processes leads to rational decisions in a standard probabilistic inference task that does not mislead people or cause them to overlook relevant aspects remains an open question. The tasks we use in the current study make all information immediately available, allowing individuals to rely on a combination of System 1 and System 2 processes. System 1 processes may be sufficient to solve these tasks if a coherence-based process accurately integrates probabilistic information. Deliberate interventions based on System 2 processes could either distort a well-calibrated System 1 process, thereby causing harm, or correct a faulty System 1 process. Hence, a higher tendency for cognitive reflection may be either helpful or detrimental in such tasks (see also Phillips et al., 2016).

It is of particular interest whether individuals’ general tendency to activate deliberate processes (i.e., CRT as a trait) leads to the same effects as those observed when decision mode is manipulated experimentally via instruction in this class of tasks (Forst & Glöckner, 2026).

### 1.4. The Cognitive Reflection Test (CRT) as a Measurement Tool

While researchers have widely used the CRT as an indicator of deliberate or reflective thinking, several limitations have been noted (for a recent review, see Sirota et al., 2021). First, the CRT appears to capture both dispositional reflection tendencies and cognitive ability (e.g., intelligence and numeracy), making it difficult to disentangle reflective processing from general cognitive capacity. Cognitive reflection as measured by the CRT and measures of cognitive abilities are highly correlated (e.g., Frederick, 2005). Furthermore, response times to CRT items (which should indicate reflection) and the accuracy of responses are positively related, but only at a relatively modest level (*r* = 0.18) and not consistently across all items (Stupple et al., 2017). Second, the widespread use of the original three-item version of the CRT has led to increased item familiarity, which is associated with improved performance (Bialek & Pennycook, 2018). Third, due to its small number of items, the CRT has a relatively low reliability (Baron et al., 2015). To address these concerns, we include an independent measure of intelligence in our analyses to control for variance attributable to cognitive ability. In addition, we employ the longer seven-item version of the CRT (Toplak et al., 2014), which demonstrates improved psychometric properties and includes less widely known items.

More generally, because the CRT relies exclusively on behavioral outcomes (e.g., response choice and accuracy), researchers must draw inferences about the engagement of reflective versus intuitive processing—and, based on these, about individuals’ general tendencies—with caution. In the CRT, multiple underlying cognitive processes can produce the same response, which limits the extent to which researchers can derive process-level conclusions and, even more so, trait inferences from the observed outcome data alone.

## 2. Hypotheses

Glöckner et al. (2024) collected more than 60,000 choices in probabilistic inference tasks and fitted machine learning models (i.e., neural networks) to investigate whether existing theories approximate their performance. For the current research, we combined these data with a second data set that contains a comprehensive assessment of participants’ personality and cognitive abilities, including the 7-item version of the CRT (Toplak et al., 2014), as well as decisions in risky choice tasks. This allowed us to investigate more detailed research questions concerning cognitive processes and interindividual differences. Specifically, we test the following partially adversarial hypotheses:

**H1a/b.**  *Rational choices in probabilistic inference tasks (i.e., choices in line with naïve Bayes) increase/decrease with CRT score.*

We used adversarial hypotheses for H1 because, from a theoretical perspective, it was a priori unclear whether positive or negative effects of cognitive reflection should prevail in tasks that people typically solve intuitively, given established effects of a match/mismatch between thinking styles and demands of decision tasks (cf. Phillips et al., 2016).

We also test effects of CRT on response time and confidence. Evidence accumulation implementations of dual-process models could account for differences in cognitive reflection by assuming that individuals with higher CRT scores have a lower deliberation threshold and a higher decision threshold. That is, they activate deliberate reflection even in less ambivalent situations and make choices only after collecting stronger evidence that one option is better than the other(s). Everything else being equal, this implies that individuals with higher CRT scores should require more time in these judgment tasks. PCS-DM does not make a clear prediction concerning the main effect of CRT on response time but provides more nuanced predictions that are tested in further exploratory analyses described below.

**H2.**  *Average response times in the probabilistic inference tasks increase with individuals’ CRT scores.*

Under the assumptions described above, evidence accumulation accounts of dual processing predict that individuals with higher CRT scores should report greater confidence in their choices, because they accumulate evidence up to a higher desired level of confidence (i.e., the evidence threshold). Conversely, intuition-based responses are also sometimes assumed to produce particularly high confidence (but see De Neys et al., 2011, 2013, for opposite findings). Forst and Glöckner (2026), in turn, found no confidence differences between intuition and deliberation instructions. This result conflicts with evidence accumulation predictions but is fully consistent with PCS-DM, which does not assume that more reflective individuals use higher evidence thresholds and therefore does not predict systematic confidence differences as a function of cognitive reflection. Because the predictions are not entirely clear, we test adversarial hypotheses:

**H3a/b.**  *Confidence in choices in probabilistic inference tasks increases/decreases with CRT score.*

In further analyses, we aim to replicate earlier findings about the relation of CRT scores and response time (H4) and on the relation of CRT on aspects of rationality in a different domain (H5–H7), such as consistency in risky choices measuring risk and loss aversion:

**H4.**  *CRT scores increase with response time when answering CRT questions.*

**H5.**  *Risk aversion (Holt & Laury, 2002, list method) decreases with increasing CRT scores.*

**H6.**  *Loss aversion (Gächter et al., 2022, list method) decreases with increasing CRT scores.*

**H7.**  *Consistency in risk aversion and loss aversion increases with CRT score.*

We also explore the interrelations of CRT and the six-factor HEXACO personality model (Ashton & Lee, 2007), and how both relate to task performance in probabilistic inference tasks. Conscientiousness, in particular, captures aspects of self-control and impulsivity and therefore conceptually overlaps with individuals’ tendency for cognitive reflection measured by the CRT (Ashton & Lee, 2007; Zettler et al., 2020). Within this factor, the facet diligence reflects individuals’ tendency to work hard, perfectionism captures the extent to which individuals carefully check for mistakes and think thoroughly, and prudence reflects a tendency to deliberate carefully and to inhibit impulses. On theoretical grounds, we expect these facets to be positively related to CRT, and that the shared variance between CRT, these facets, and intelligence accounts for individuals’ increased performance:

**H8a–c.**  *CRT scores correlate positively with the facets (a) diligence, (b) perfectionism, and (c) prudence of the conscientiousness dimension of the HEXACO model.*

**H9.**  *The effect of CRT on rational choice task performance (see H1) is driven by shared variance with intelligence and the facets diligence, perfectionism, and prudence of the conscientiousness dimension of the HEXACO model.* 1

If CRT reflects a stronger spontaneous tendency to deliberate, results concerning strategy use should be comparable to those from manipulations of decision mode, as implemented by Forst and Glöckner (2026). We therefore additionally investigate, on an exploratory basis:

E1: Is individuals’ choice behavior (choices, response times, and confidence ratings) more in line with the predictions of PCS-DM among individuals with higher (compared to lower) CRT scores?

E2: Is CRT related to the use of different decision strategies? Specifically, do individuals with lower CRT scores exhibit choices that are more consistent with simpler heuristics, such as take-the-best or equal weight heuristic, which ignore parts of the available information?

## 3. Method

Pre-registration, data, code, and materials for this study are available at https://osf.io/hdasm/ (accessed on 10 May 2026).

### 3.1. Participants and Design

We reanalyzed the data from Glöckner et al. (2024; data and materials of the original study are available at https://osf.io/fa4jw/, accessed on 10 May 2026) with 250 participants (after exclusions), applying the same exclusion criteria as in the original study. Specifically, we included only participants who completed this study. We excluded two participants who were correct on fewer than 55% of the choices and one individual who indicated that they did not respond seriously.

Additionally, in line with the pre-registration, we excluded one participant with an extreme outlier response time in the Cognitive Reflection Test (M+5sd) and excluded choice trials with extreme outlier response times (M+5sd). Each participant completed 250 choices in randomly generated probabilistic inference tasks and received performance-contingent incentives. The final sample comprised *N* = 249 participants (64% female, mean age = 30.7 years, overall exclusion rate of participants: 1.5%) who completed a total of *n* = 61,604 choices. Participants were recruited from the local participant pool (Decision Lab Cologne, DLC), which consists of individuals who signed up to take part in studies using hroot (Bock et al., 2014). Participants in this pool are older and more diverse than typical student samples. Our sample included 73% students and 27% non-student participants from the general public. The experiment lasted approximately 35 min. Participants received a 2 € show-up fee (approx. 2.16 USD) and performance-contingent payment of 0.04 € per correct naïve Bayesian solution (average total earnings = 10.55 €, approx. USD 11.43). An a priori sensitivity analysis (using G*Power, Release 3.1.9.7, Faul et al., 2007) indicated that the sample size allowed detecting small correlations of *rho* = 0.12 with power 1 − *beta* = 0.95.

### 3.2. Materials and Procedure

#### 3.2.1. Probabilistic Inferences Task 

The task involved choices between two options based on predictions from four probabilistic cues presented openly to participants. Cue validities (e.g., 78 of 100 correct) and cue predictions (i.e., “+” vs. “−” for a prediction of good vs. bad performance of the respective stock) for the options (Stock A or B) differed across the 250 trials. An example trial is shown in Figure 1. Response time was measured from trial onset until the mouse click selecting an option. On a second screen, participants indicated their choice confidence on a scale from very uncertain (50) (i.e., random) to very certain (100). A choice was coded as rational when it aligned with naïve Bayes. For the main analyses we collapsed choices, response times, and confidence ratings to average scores per person (i.e., *p* (rational), average decision time, and average confidence). The full materials in German and an English translation are available on OSF. Further procedural details are reported in Glöckner et al. (2024). For the present re-analysis, we merged data from this choice task with data from a base assessment that all participants completed online upon joining the DLC subject pool. All subsequent measures are drawn from this base assessment.

#### 3.2.2. Cognitive Reflection 

Cognitive reflection was measured using a 7-item version of the Cognitive Reflection Test from Toplak et al. (2014), which includes the three original items (Frederick, 2005). The items are designed such that they elicit an intuitive but incorrect response that must be overridden through reflection. As the dependent measure, we coded a CRT7 score as the number of correct responses, with higher scores indicating a stronger tendency for cognitive reflection. We additionally recorded the total response time for completing all seven items. As a robustness check, we repeated the analyses using an alternative score capturing the number of intuitive responses, the CRT intuition score, which yielded the same conclusions.

#### 3.2.3. Risk and Loss Aversion

We measured risk aversion using a standard list method (Holt & Laury, 2002). In this list, participants repeatedly chose between a relatively safe lottery and a risky lottery, with the risky lottery gradually increasing in attractiveness (i.e., expected value). Three examples are: Decision 4: (4/10, 2.00 €; 6/10, 1.60 €) vs. (4/10, 3.85 €, 6/10, 0.10 €); Decision 5: (5/10, 2.00 €; 5/10, 1.60 €) vs. (5/10, 3.85 €, 5/10, 0.10 €); Decision 6: (6/10, 2.00 €; 4/10, 1.60 €) vs. (6/10, 3.85 €, 4/10, 0.10 €).

In the 10 trials, the probabilities for the first outcome of each option varied from 1/10 to 10/10. The number of choices of the safe lottery (out of 10) served as the measure for risk aversion. Individuals with consistent risk preferences should switch exactly once from the safe to the risky lottery. Consistency in risky lotteries is coded 1 if there was a single switching point (and otherwise 0). Loss aversion was measured using a similar list method (Gächter et al., 2022). Individuals decided whether to accept or reject a series of 50:50 lotteries involving a gain of 3 € and a gradually increasing loss (from 1.00 € to 3.50 €). Individuals with consistent loss preferences should switch exactly once (or never) from accepting to rejecting these lotteries (i.e., consistency loss aversion = 1, otherwise 0). We computed loss aversion only for individuals with consistent choices. If participants rejected all lotteries, their loss aversion exceeded 3 (coded as 3.5). If they accepted all lotteries, their loss aversion was below 0.87 (indicating loss seeking, coded as 0.37). If they switched, e.g., from accept to reject between a loss of 1 € and 1.5 €, their loss aversion lay between 3 and 2. We used the midpoint of the corresponding interval as the estimate of individuals’ loss aversion (and values +/−0.5 for the boundary categories as described above). The overall consistency score for risk and loss aversion was computed as the sum of both consistency indicators. Choice behavior in both measures was incentivized by randomly selecting one decision for payment (or potential deduction from the base payment for the baseline assessment).

#### 3.2.4. Intelligence and Personality

We measured intelligence using the 16-item ICAR 16 questionnaire (Condon & Revelle, 2014), which assesses general intelligence with 16 items from four categories (i.e., three-dimensional rotation, letter and number series, verbal reasoning, and matrix reasoning). Personality was measured using the German 104-item version of the HEXACO Personality Inventory Revised (Lee & Ashton, 2006; see also Zettler et al., 2011). For theoretical reasons, we focused on three facets of the conscientiousness factor. The diligence facet captures a tendency to work hard and maintain a strong work ethic. The perfectionism facet reflects the tendency to be thorough, detail-oriented, and careful about avoiding mistakes. The prudence facet measures the tendency to deliberate carefully, inhibit impulses and consider options carefully. Both intelligence and these personality facets are expected to capture aspects related to cognitive reflection.

## 4. Results

Descriptive statistics, intercorrelations, and reliabilities for all relevant variables and scales are reported in Table A1 in the Appendix A. Importantly, the reliability of the CRT7 score was in the acceptable range (Cronbach’s *alpha* = 0.70) and its correlation with intelligence was high (*r* = 0.62).

**Figure 2:**
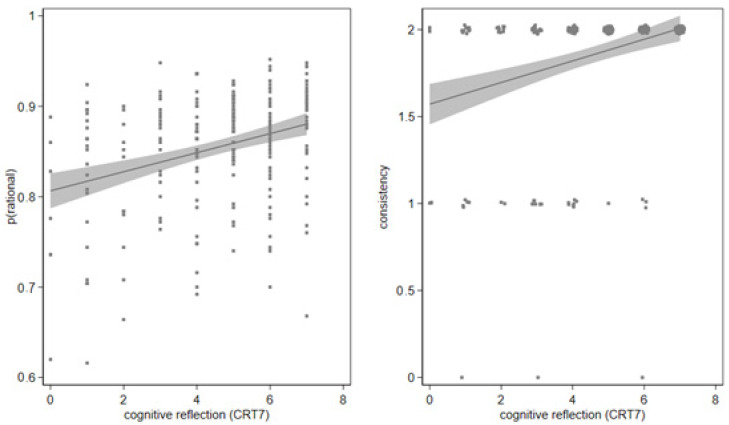
Rationality as a function of cognitive reflection: proportion of rational choices in probabilistic inference task (**left**) and consistency in risk and loss aversion measures (**right**).

Concerning our core research questions, we found that, in incentivized probabilistic inference tasks, the proportion of rational choices in line with naïve Bayes increases with individuals’ cognitive reflection (*r* = 0.32, *p* < 0.001, Figure 2, left). A regression analysis controlling for intelligence, age, and sex showed that this relation is partially—though not fully—accounted for by intelligence. Specifically, intelligence emerged as a strong predictor (*beta*^2^ = 0.28, *t*(244) = 3.79, *p* < 0.001), while the effect of cognitive reflection was reduced by more than half (*beta* = 0.14, *t*(244) = 1.86, *p* = 0.063).

An analysis of decision times in choices did not support the hypothesis that response times increase with individuals’ tendency for cognitive reflection (*r* = −0.03, *p* = 0.68). A regression analysis controlling for intelligence revealed an unexpected suppression effect, contrary to H2. Specifically, decision times increased with intelligence (*beta* = 0.39, *t*(244) = 5.02, *p* < 0.001) but decreased with increasing cognitive reflection (*beta* = −0.24, *t*(244) = 3.15, *p* = 0.002). Because our analyses are purely correlational, these findings do not imply causal effects. Overall, we found no evidence that cognitive reflection is associated with longer decision times in these tasks. We also did not observe a systematic association between cognitive reflection and confidence in choices (*r* = 0.03, *p* = 0.59, cf. H3). Both null findings for H2 and H3 are theoretically important. They suggest that integrative dual-process models relying on standard evidence accumulation mechanisms may not adequately capture the underlying processes. Such models would predict longer response times and higher confidence among individuals more inclined to deliberate and to avoid uncertainty, as these individuals should apply higher decision thresholds.

We did not replicate the previously reported (*r* = 0.18, Stupple et al., 2017) positive—albeit small—correlation between cognitive reflection and response time in answering CRT items (*r* = 0.08, *p* = 0.19, i.e., no support for H4). We also did not replicate the previously reported negative relations between CRT and risk aversion (*r* = −0.05, *p* = 0.39, i.e., no support for H5) or loss aversion (*r* = −0.002, *p* = 0.98, *N* = 234, i.e., no support for H6).

In line with H7, we observed that consistency in the risk aversion and loss aversion measures increased with cognitive reflection (*r* = 0.32, *p* < 0.001, Figure 1, right). Most participants exhibited consistent choices, but inconsistent behavior was more frequent among individuals with lower cognitive reflection.

Contrary to H8, we found no evidence for a relation between cognitive reflection and the conscientiousness facets diligence (*r* = 0.05, *p* = 0.44), perfectionism (*r* = 0.09, *p* = 0.14), or prudence (*r* = 0.05, *p* = 0.41). This finding was unexpected, given the conceptual overlap implied by their definitions.

The effect of cognitive reflection on the proportion of rational choices in probabilistic inference tasks was no longer statistically significant in a two-sided test (*beta* = 0.14, *t*(243) = 1.89, *p* = 0.059) when controlling for intelligence, diligence, perfectionism, and prudence (Table A2). However, the results indicate that the association between CRT and rational choices is not fully accounted for by shared variance with these factors. Thus, the findings are only partially consistent with H9, which posited that the relation between CRT scores and rational choice is driven by shared variance with intelligence and the personality facets.

### Exploratory Analyses

In exploratory analyses, we examined whether differences in CRT scores were reflected in qualitative differences in processing. To control for alpha error accumulation due to multiple testing, we applied a Bonferroni corrected significance threshold of alpha = 0.05/6 = 0.0083 in these analyses (i.e., four tests for E1 and two tests for E2). First, we tested whether individuals with higher cognitive reflection showed decision behavior more consistent with PCS-DM predictions, as recently observed in a study manipulating decision mode (Forst & Glöckner, 2026). We focus on results for PCS-DM(fitted), a version of PCS-DM with one free parameter per person that was fitted to the data. This parameter *P* captures interindividual differences in sensitivity to cue validities. The one-parameter model has shown the best performance in predicting behavior in previous work (e.g., Glöckner et al., 2024). Importantly, the general pattern of results also holds when using PCS-DM without a free parameter (i.e., with the parameter fixed to its default value of *P* = 1.9).

**Figure 3:**
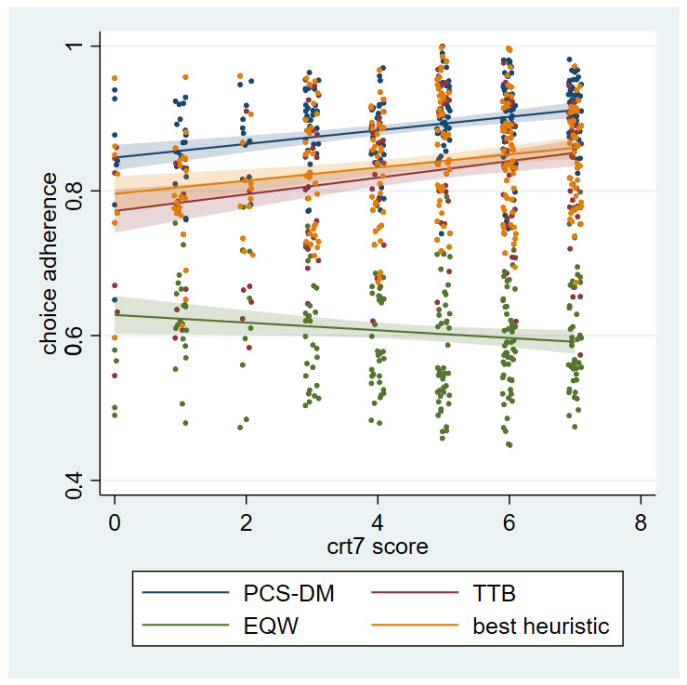
Proportion of choices consistent with the predictions of different strategies as a function of cognitive reflection. The strategies include Parallel Constraint Satisfaction for Decision Making (PCS-DM) (with one fitted sensitivity parameter per person), take-the-best heuristic (TTB), equal weight heuristic (EQW), and “best heuristic” (i.e., for each participant, the better-performing heuristic among TTB and EQW). Lines represent regression fits with 95% CIs.

As expected, choice adherence to PCS-DM(fitted) predictions was high across all levels of cognitive reflection (Figure 3, blue line) and increased substantially with CRT scores (Table A3, model 1; *p* < 0.001; support for E1). Specifically, individuals with the lowest cognitive reflection scores (CRT score = 0) showed 84% of choices in line with PCS-DM predictions, whereas individuals with maximal cognitive reflection (CRT score = 7) showed 91% adherence. This relation was substantially attenuated and remained significant only at the uncorrected alpha error level when controlling for interactions with intelligence and the conscientiousness facets diligence, perfectionism, and prudence in the regression (*p* = 0.049). Across all levels of cognitive reflection above zero, individuals showed substantial correlations between predicted and observed decision time (all *r* > 0.38 and *r* < 0.60). Individuals higher in cognitive reflection exhibited decision times more consistent with PCS-DM predictions, as indicated by a significant interaction (Table A3, model 2, *p* = 0.016), although this effect did not remain significant after Bonferroni correction. For confidence, correlations between predicted and observed values were also substantial for all levels of cognitive reflection above zero (all *r* > 0.45 and *r* < 0.58), but the interaction effect did not reach conventional significance levels (Table A3, model 3). Individuals with minimal cognitive reflection (CRT score = 0) showed markedly lower correlations for response time (*r* = 0.07) and confidence (*r* = 0.17) compared to all other CRT levels. However, these estimates should be interpreted cautiously, as only seven participants fell into the former category, resulting in less stable estimates.

Second, we examined whether individuals low in cognitive reflection relied more on simple heuristics that ignore parts of the information (i.e., the take-the-best heuristics or the equal weight heuristic, E2). To this end, we compared individuals’ adherence to the choice predictions of different strategies as a function of CRT (Figure 3). We found no evidence that individuals with lower CRT scores showed greater adherence to heuristic predictions. For both TTB and a “best heuristic” (i.e., the better-fitting of TTB or EQW per individual), we observed a significant positive relation with CRT (both *beta* = 0.23, *t* > 3.6, *p* < 0.001). Thus, there is no indication of a qualitative difference in strategy use between participants with low vs. high CRT scores in the theoretically expected direction.

**Figure 4:**
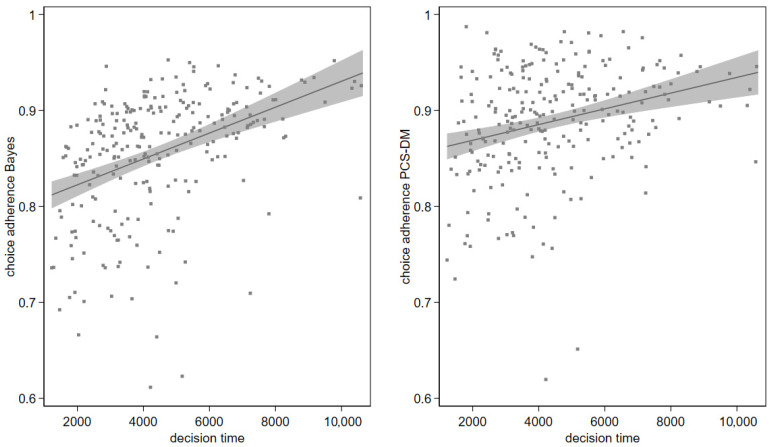
Relation between decision time on adherence of choices to the rational solution according to Naive Bayes (**left**) and PCS-DM (**right**).

One further notable finding was that individuals’ overall performance (i.e., the proportion of rational responses) correlated strongly and positively with decision time (Table A1, Figure 4, left, *r* = 0.40). Thus, correct (rational) responses tended to require more time than incorrect ones. Similarly, individuals with higher average decision times showed choices that were more consistent with the predictions of PCS-DM (Figure 4, right, *r* = 0.28). Although no causal claims can be drawn from these correlational findings, they are informative with respect to potential process assumptions. The correlations are consistent with the assumption that more careful application of a decision strategy and more thorough construction of the mental representation may enhance performance and consistency. Notably, while performance increased with CRT scores (H1), decision time did not (H4), suggesting that the association between CRT and performance is not driven by longer deliberation per se. Instead, the positive effects of higher CRT and longer decision times on performance appear to be largely independent. One possibility is that individuals with higher CRT scores use available time more efficiently to improve performance. A regression analysis including decision time, CRT and intelligence as predictors showed that all three factors independently contributed to predicting individual performance and jointly explained 29% of the variance (Table A4).

Overall, PCS-DM predicted decision making well for both more reflective and more intuitive individuals and outperformed competing models. Across tasks, the higher proportion of rational choices among individuals with higher cognitive reflection was neither accompanied by longer decision times nor by increased confidence. Moreover, the increased rationality was not driven by qualitative shifts in decision strategies (e.g., greater reliance on simple heuristics among individuals low in CRT). The only notable difference was that highly reflective individuals showed choices (and, as a trend, also response times) more consistent with PCS-DM predictions. This pattern suggests that individuals with high and low cognitive reflection rely on the same underlying processes. However, highly reflective individuals appear to apply these processes more efficiently and more consistently.

## 5. Discussion

Previous research has shown that higher cognitive reflection is associated with fewer biased choices and more rational responses across a range of tasks. However, most studies have focused on paradigms specifically designed to elicit biased responses by potentially misleading participants. In such tasks, cognitive reflection may facilitate the detection of biasing influences and the deliberate correction of initial responses. In the present study, we examined whether individuals’ tendency toward higher cognitive reflection is associated with more or less rational choices in standard probabilistic inference tasks, in which such corrective processes are not required. At the theoretical level, we investigated whether potential differences in rationality are accompanied by qualitative shifts in decision strategies, as assumed by classic dual-process models. As an alternative, we considered integrated dual-process models, which posit that the same underlying processes govern both intuitive and deliberate cognition. Consistent with this perspective, a recent study manipulating decision mode via instruction (Forst & Glöckner, 2026) found no qualitative shifts in decision strategies. Instead, a Parallel Constraint Satisfaction model for decision making (PCS-DM) provided the best account of behavior under both decision modes, supporting integrated dual-process models.

The current study shows that individuals with higher cognitive reflection make more rational choices in non-biasing tasks. The results hold for both probabilistic inferences and risky choices. For the latter, we used inconsistencies in responses as a proxy for irrationality. The effect of cognitive reflection on rationality was partially, but not fully, explained by shared variance with intelligence. An additional exploratory analysis indicated that cognitive reflection, intelligence, and average decision time independently contributed to predicting individual performance in probabilistic inference tasks. These findings suggest that performance is shaped by multiple factors related to task comprehension: (a) general cognitive abilities captured by intelligence, (b) aspects of cognitive reflection that extend beyond intelligence as measured by the CRT, and (c) motivational factors reflected in greater time investment and more careful processing, which go beyond cognitive reflection per se.

Decision time and confidence did not increase with cognitive reflection. These null effects are informative and of high theoretical relevance. They speak against explanations based on (a) standard implementations of evidence accumulation models and (b) heuristic models that could potentially explain the observed increase in rational choices among individuals with higher cognitive reflection. First, the null findings challenge integrative dual-process models that explain interindividual differences in cognitive reflection (and differences between System 1 and System 2 processes more generally) in terms of variation in evidence thresholds within standard evidence accumulation models. If highly reflective persons mainly differed from those with low reflection in having a higher evidence threshold, they should necessarily exhibit longer response times and higher confidence levels. Because we did not observe this pattern, such implementations of integrative dual-process evidence accumulation models are not supported by the data. Our data further show that lower cognitive reflection is not necessarily associated with the use of simple heuristics that ignore parts of the information and therefore potentially lead to suboptimal results.

In line with Forst and Glöckner (2026), our results suggest that a third alternative, namely coherence-based mechanisms as formalized in PCS-DM, best accounts for the behavior of both less and more reflective individuals. Across all levels of cognitive reflection, participants showed high adherence of choices to PCS-DM predictions. Predictions for decision times and confidence were also strongly correlated with the observed data as for all levels of cognitive reflection above zero. Individuals with higher CRT scores exhibited behavior closely aligned with the model than those with low CRT scores. This pattern was particularly pronounced for choice behavior and, as a trend, also emerged for decision times, but not for confidence. Moreover, individuals who spent more time on their decisions showed choices that were both more consistent with PCS-DM and more rational. From the perspective of PCS models, one potential explanation is that individuals with higher CRT scores apply the same underlying processes more efficiently and consistently. Specifically, they may translate available information into mental representations more reliably and/or implement coherence-maximizing processes with fewer errors. The results further suggest that these differences are partly attributable to general cognitive abilities (e.g., intelligence, numeracy) and (perhaps to a lesser extent) to differences in cognitive reflection per se—both of which contribute to CRT scores. In addition, there appear to be independent effects of careful processing, as reflected in longer decision times.

It should be noted that most of our conclusions regarding process models are based on post hoc exploratory analyses and that our study design was correlational. Further confirmatory and experimental studies are required to critically test the interpretations presented here. It is possible that refined dual-process models assuming distinct processes or different implementations of evidence accumulation models could account for the present data as well as, or even better than, the models considered in this discussion. Moreover, it is plausible that, under certain conditions, reasoning operates in a structurally different manner rather than differing only in degree of efficiency within the same underlying processes. Accordingly, the conclusions of the current study should be generalized to other tasks only with caution. Further research is needed to delineate the boundary conditions of integrated dual-process models such as those discussed in this paper.

The observation that behavior across all levels of cognitive reflection closely followed the predictions of PCS-DM, coupled with the absence of qualitative differences in processing between individuals with low versus high CRT scores, suggests that differences in processing in probabilistic inference tasks are more gradual than one might have expected a priori. Accordingly, our findings provide preliminary support for integrated dual-process models. Implementations of such models based on interactive activation mechanisms, as proposed in PCS-DM, appear to account well for the observed data and are further supported by the present results.

### 5.1. Further Findings

We did not replicate previous findings linking CRT to response time when answering CRT items. Likewise, we found no association between CRT and risk or loss aversion (Frederick, 2005). One possible explanation is that our approach allowed us to separate consistency in these measures from the underlying preference parameters, but further research is needed to evaluate this possibility.

We found no correlations between cognitive reflection and the HEXACO conscientiousness facets of diligence, perfectionism, and prudence. This result is inconsistent with theoretical expectations, as the facets are assumed to capture core aspects of cognitive reflection. This divergence between standard questionnaire measures and behavioral measures is therefore notable—although consistent with findings in related domains (Frey et al., 2017)—and warrants further investigation into its underlying causes.

One limitation of our work is that we did not measure numeracy. Numeracy refers to individuals’ ability to process numerical and probabilistic information and is often correlated with CRT scores (Sirota & Juanchich, 2011). We therefore cannot rule out that the effect of cognitive reflection on rationality is partly driven by numeracy. However, because numeracy is also strongly correlated with intelligence, our intelligence measure likely captured at least those aspects of numeracy that share variance with it. At the same time, numeracy may facilitate faster task performance by enabling individuals to grasp the gist of the situation more quickly, potentially counteracting the effects predicted in H2 (see also Reyna et al., 2009).

### 5.2. Conclusions

Cognitive reflection increases the likelihood of rational choices even in tasks that are not designed to mislead participants. This holds for both probabilistic inference tasks, in which Bayes provides a natural criterion of rationality, and risky choice tasks, in which rationality is operationalized as choice consistency. There is no indication that the superior performance of individuals with higher CRT scores is due to the use of qualitatively different decision strategies or greater time investment. Rather, across all levels of cognitive reflection (albeit somewhat less so for individuals with CRT scores of zero), behavior is best described by the same PCS-DM model. Individuals with higher CRT scores appear to rely on the same coherence-structuring processes as those with lower CRT scores but apply them more consistently, as reflected in a closer alignment between their behavior and model predictions. Overall, our findings support an integrative perspective on dual-process models, particularly those based on interactive activation mechanisms (McClelland, 2010; McClelland et al., 2014).

## Data Availability

Data, code, and materials are available at https://osf.io/hdasm/ (accessed on 10 May 2026).

## Notes

1 In the original formulation of this hypothesis, we predicted a mediation. As an anonymous reviewer noted, assuming causal mediation is not appropriate in this context. We therefore refer to what we originally intended: assessing the extent to which the relation is accounted for by shared variance with the respective factors. The mediation results lead to the same corresponding conclusions.

2 Here and in the following, beta refers to standardized regression weights (i.e., partial correlations).

## Appendix A

**Table A1:** Descriptive statistics and correlations.

Measures	M	sd	1	2	3	4	5	6	7	8	9	10	11	12	13
1. CRT7 score (0–7)	4.57	1.97	(0.70)												
2. *p* (rational) (0–1)	0.85	0.06	0.32 ***	(0.91)											
3. decision time (sec)	4.45	2.00	−0.03	0.40 ***	-										
4. confidence (50–100)	82.1	7.93	0.03	0.13 *	−0.12	-									
5. time CRT (sec)	474.2	332.66	0.08	0.08	0.24 ***	0.07	-								
6. risk aversion (0–10)	5.49	1.67	−0.05	−0.05	−0.14 *	−0.02	−0.04	-							
7. loss aversion (0.37–3.5)	1.73	0.66	0.00	−0.01	−0.05	−0.07	0.11	0.24 ***	-						
8. consistency (0/1/2)	1.86	0.39	0.32 ***	0.24 ***	0.05	0.07	0.02	0.04	0.04	-					
9. IQ ICAR16 (0–1)	0.62	0.21	0.62 ***	0.39 ***	0.22 ***	0.09	0.11	−0.07	−0.06	0.30 ***	(0.70)				
10. diligence (1–5)	3.80	0.82	0.05	0.04	0.02	0.00	0.04	0	−0.03	0.06	0.01	(0.77)			
11. perfectionism (1–5)	3.63	0.77	0.09	0.07	0.12	−0.11	0.06	0.03	0.02	0.15 *	0.12	0.53 ***	(0.71)		
12. prudence (1–5)	3.55	0.72	0.05	0.12	0.05	−0.07	−0.06	0.10	0.06	0.04	0.12	0.45 ***	0.39 ***	(0.66)	
13. female (0/1 = yes)	0.64	0.48	−0.17 **	−0.08	0.11	−0.14 *	0.05	0.04	0.17 *	−0.09	−0.04	0.14 *	0.15 *	0.04	
14. age (years)	30.68	11.61	−0.02	−0.21 ***	0.10	−0.03	0.09	−0.12	−0.04	−0.05	−0.11	−0.05	−0.09	−14 *	−0.09

Note. Scale reliabilities (Cronbach’s alpha) are provided in the diagonal in parentheses, where available. Constructs are coded as indicated in parentheses in the first column; unless noted otherwise, higher scores indicate higher levels on the respective variable/construct. The effective total sample size is *N* = 249. *** *p* < 0.001, ** *p* < 0.01, * *p* < 0.05.

**Table A2:** OLS regression predicting individuals’ rational choices in probabilistic inference tasks from cognitive reflection, controlling for intelligence and personality facets (test of H9). The criterion variable is *p* (rational) computed at the participant level.

	(1)	(2)	(3)	(4)
CRT7 score	0.011 ***	0.004 ^+^	0.010 ***	0.005 ^+^
IQ ICAR16		0.092 ***		0.089 ***
diligence			−0.002	0.000
perfectionism			0.001	−0.001
prudence			0.010	0.008
constant	0.806 ***	0.777 ***	0.777 ***	0.754 ***
*N*	249	249	249	249
ll	345.545	353.643	347.176	354.614
aic	−687.091	−701.286	−684.352	−697.227
bic	−680.056	−690.733	−666.765	−676.123

*** *p* < 0.001, ^+^ *p* < 0.10.

**Table A3:** Regression analysis predicting behavior using PCS-DM(fitted).

	(1) Choice		(2) Time		(3) Confidence	
PCS choice predictions	4.1961	***				
	(0.0720)					
CRT7 score	−0.0004					
	(0.0109)					
PCS choice * CRT7	0.1841	***				
	(0.0384)					
CRT7 score			−0.0012			
			(0.0015)			
PCS time prediction			0.0065	***		
			(0.0002)			
PCS time * CRT7			0.0003	*		
			(0.0001)			
CRT7					0.1304	
					(0.2822)	
PCS confidence pred					128.9581	***
					(6.4797)	
PCS conf * CRT7					6.1214	
					(3.4387)	
Number of observations	61,604		61,604		61,604	
Number of clusters	249		249		249	
Pseudo *R*-squared	0.50					
*R*-squared			0.06		0.05	

* *p* < 0.05, *** *p* < 0.001. Note. Standard errors in parentheses and clustered by subject. All variables are centered; intercepts are not reported. PCS-DM(fitted) predictions refer to the version with one sensitivity parameter *P* fitted per participant, as in Glöckner et al. (2024).

**Table A4:** OLS regression predicting individuals’ rational choices in probabilistic inference tasks from cognitive reflection, intelligence and average decision time. The criterion variable is *p* (rational) computed at the participant level.

	Performance *p* (rational)
CRT7 score	0.0076 ***
IQ ICAR16	0.0494 *
decision time (in sec)	0.0119 ***
constant	0.7360 ***
*N*	249
*R* ^2^	0.29

*** *p* < 0.001, * *p* < 0.05.
